# Development of a New Tomato Torrado Virus-Based Vector Tagged with GFP for Monitoring Virus Movement in Plants

**DOI:** 10.3390/v12101195

**Published:** 2020-10-20

**Authors:** Przemysław Wieczorek, Marta Budziszewska, Patryk Frąckowiak, Aleksandra Obrępalska-Stęplowska

**Affiliations:** Department of Molecular Biology and Biotechnology, Institute of Plant Protection—National Research Institute, Władysława Węgorka 20St, 60-318 Poznań, Poland; m.budziszewska@iorpib.poznan.pl (M.B.); p.frackowiak@iorpib.poznan.pl (P.F.)

**Keywords:** tomato torrado virus, sGFP, plant pathology, infectious clone, plant-virus interaction

## Abstract

Green fluorescent protein (GFP)-tagged viruses are basic research tools widely applied in studies concerning molecular determinants of disease during virus infection. Here, we described a new generation of genetically stable infectious clones of tomato torrado virus isolate Kra (ToTV_pJL_-Kra) that could infect *Nicotiana benthamiana* and *Solanum lycopersicum*. Importantly, a modified variant of the viral RNA2—with inserted sGFP (forming, together with virus RNA1, into ToTV_pJL_-Kra_GFP_)—was engineered as well. RNA2 of ToTV_pJL_-Kra_GFP_ was modified by introducing an additional open reading frame (ORF) of sGFP flanked with an amino acid-coding sequence corresponding to the putative virus protease recognition site. Our further analysis revealed that sGFP-tagged ToTV-Kra was successfully passaged by mechanical inoculation and spread systemically in plants. Therefore, the clone might be applied in studying the in vivo cellular, tissue, and organ-level localization of ToTV during infection. By performing whole-plant imaging, followed by fluorescence and confocal microscopy, the presence of the ToTV_pJL_-Kra_GFP_-derived fluorescence signal was confirmed in infected plants. All this information was verified by sGFP-specific immunoprecipitation and western blot analysis. The molecular biology of the torradovirus-plant interaction is still poorly characterized; therefore, the results obtained here opened up new possibilities for further research. The application of sGFP-tagged virus infectious clones and their development method can be used for analyzing plant-virus interactions in a wide context of plant pathology.

## 1. Introduction

In vivo monitoring of virus movement, as well as its subcellular localization in an infected host plant, can answer many important questions at the forefront of modern molecular plant virology. Currently, it is possible to characterize virus localization in tissues using a wide range of monitoring approaches (reviewed in [1]). Nevertheless, it is beneficial to use in vivo monitoring techniques based on labeling the target virus with a small nontoxic and easily detectable marker. Currently, green fluorescent protein (GFP) seems to be the gold standard in studies focusing on expressing foreign genes from virus genomes [2]. For this purpose, a wide range of virus-based expression systems were developed and applied either in plants [3,4,5] or animal systems [6].

*Tomato torrado virus* (ToTV) is a type member of the *Torradovirus* genus within the *Secoviridae* family. ToTV efficiently infects *Solanum lycopersicum*, inducing severe necrosis in tomato and resulting in plant crop loss. Other plant species were also previously described as hosts for ToTV [7], including *Nicotiana benthamiana*, a well-characterized model organism widely used in plant-pathogen interaction studies [8]. Tomato torrado virus is transmitted by whiteflies: *Trialeurodes vaporariorum*, *T. abutilonea* and *Bemisia tabaci* [9]. ToTV, similar to other torradoviruses, has a bipartite single-stranded genome and consists of RNA1 (7829 nt) and RNA2 (5404 nt), tailed with a poly-A track [10]. The self-replicating RNA1 of torradoviruses [11] encodes a single long polyprotein with protease cofactor (ProCo), helicase (Hel), viral protease (Pro), and RNA-dependent RNA polymerase (RdRP) motifs. RNA2 encodes ORF1 (necessary for systemic infection [12]) overlapping the long ORF2 encoding movement protein (3A) followed by three capsid protein (CP) subunits (Vp35, Vp26, and Vp23). Recently, several pathogenicity determinants were described for ToTV [13,14,15] and tomato marchitez virus (ToMarV) [12].

In this study, we designated ToTV_pJL_-Kra, the second generation of infectious clones of ToTV (isolate Kra). In these novel clones, cDNA copies of ToTV genomic RNAs were cloned between the 35S promoter of cauliflower mosaic virus (CaMV) and hepatitis delta virus (HDV) ribozyme followed by the 35S terminator of CaMV in a low-copy pJL89 plasmid vector, which is widely used in engineering infectious clones of plant viruses [11,16,17]. Moreover, the sGFP-tagged version of ToTV RNA2 was developed, and together with ToTV RNA1, composed ToTV_pJL_-Kra_GFP_, efficiently infected *N. benthamiana* and tomato plants. During ToTV_pJL_-Kra_GFP_ infection, sGFP was produced from the RNA2-encoded polyprotein, which was confirmed by monitoring green fluorescence in plants (verified by fluorescence and confocal microscopy) and GFP-specific immunoprecipitation (IP) and western blotting. The use of the sGFP-tagged infectious clone allows the monitoring of ToTV transport in the entire plant as well as its localization in infected cells. The sGFP-tagged infectious clone of ToTV, together with the enclosed protocol of production thereof, can be applied for engineering similar constructs for all other members of the *Torradovirus* genus.

## 2. Materials and Methods

### 2.1. Plasmid Construction

All the described genetic engineering manipulations were performed according to standard protocols [18]. First, the previously described full genomic copies of infectious clones of ToTV (p35Kra1 and p35Kra2) [19] were subcloned from their backbone vector (pGreen) to the destination vector pJL89 (the pJL89 plasmid was kindly provided by Professor Masimo Turina). Briefly, 20 ng of the pJL89 plasmid was PCR-amplified with CloneAmp HiFi PCR Premix (Takara, Kusatsu, Shiga, Japan) and the primer pair pJL89ToT1_R/pJL89ToT1i2_F or pJL89ToT2_R/pJL89ToT1i2_F to generate pJL89-based plasmids suitable for cloning full cDNA copies of ToTV RNA1 or RNA2, respectively (Table 1). Next, the full-length cDNA copies of the ToTV RNAs were PCR-amplified using 20 ng of template plasmids (either p35Kra1 or p35Kra2) with CloneAmp HiFi PCR Premix (Takara) and the primers asTo1A_pJL_FW/asTo2C_pJL_RV or asTo2A_pJL_FW/asTo2C_pJL_RV for RNA1 or RNA2, respectively.

One hundred nanograms of the PCR product of the plasmid backbone was mixed with 100 ng of amplified virus cDNA in the presence of 1× NEBuilder HiFi DNA Assembly Master Mix (NEB, Ipswich, MA, USA). The resulting mixture was *DpnI* treated (Thermo Scientific, Waltham, MA, USA) and transformed into *E. coli* Stellar competent cells (Takara). The resulting plasmids pJL89-Kra1 and pJL89-Kra2 were isolated from *E. coli* transformants, sequenced, and tested for their infectivity in *N. benthamiana* and *S. lycopersicum* (Betalux cultivar) (as described previously [19] and further in the paragraph). Next, pJL89-Kra2 was used as a backbone for preparing the RNA2-based expression vector. For this purpose, the additional sequence encoding the putative protease recognition site flanking the C_2132_AG/GTG_2137_ codons (encoding the Q481/V482 putative protease cleavage site between the 3A and Vp35 motifs within the long polyprotein encoded by ToTV RNA2 [20]) was introduced within pJL89-Kra2 using the primers pJLRNA2_CASF4 and pJLRNA_CASR4 and utilizing CloneAmp HiFi PCR Premix (Takara). Next, the coding sequence of sGFP was PCR amplified using the primers EGFP_CASF3 and EGFP_CASR3, and the resulting cDNA was inserted within pJL89-Kra2 using the Gibson assembly protocol. The created construct pJL89-Kra2-GFP was Sanger sequenced and used for the transformation of *Agrobacterium tumefaciens* GV3101.

### 2.2. Plant Material, Agroinfiltration, and Sap Inoculation

*Nicotiana benthamiana* and tomato (*S. lycopersicum*, Betalux cultivar) seeds were germinated in an autoclaved universal growth medium in pots in a growth chamber. When the first two true leaves were fully expanded, the seedlings were transplanted individually into 98-cell seed germination trays containing the same universal growth medium and were watered daily. After 10 days of growing, the seedlings were transplanted individually into plastic pots (10 cm in diameter) for further growth and were maintained in a greenhouse with a photoperiod and temperature of 16 h 28 °C/8 h 24 °C (day/night).

Agroinfiltration was performed as described previously [19]. Briefly, a single colony of the recombinant *A. tumefaciens* bacteria was grown in liquid LB medium (supplemented with 50 mg/L rifampicin and 100 mg/L kanamycin) at 28 °C for 48 h with shaking. Afterward, the bacteria were pelleted by centrifugation and resuspended to an OD600 = 1.0 in agroinfiltration buffer (10 mM 2-(N-morpholino)ethanesulfonic acid pH 5.6, 10 mM MgCl_2_, 200 μM acetosyringone).

Sap inoculation was performed as described by Budziszewska et al. [21]. Briefly, plant material (collected from the 3 infected plants) was ground with a mortar and pestle in the presence of 0.1 M phosphate buffer and mechanically inoculated onto carborundum-dusted leaves of tested plants.

### 2.3. Virus Detection by RT-PCR

Virus detection in systemic leaves of infected/infiltrated plants was performed utilizing RT-PCR. To achieve this aim, total RNA was isolated using TriReagent (Thermo Scientific) and precipitated with isopropanol [22]. The resulting total RNA (ca. 1 µg) was converted to cDNA using 200 U of RevertAid Reverse Transcriptase (Thermo Scientific) in the presence of 50 ng of random hexamers (Thermo Scientific). For RT-PCR, 1 µL of cDNA was used in a 20 µL reaction containing 1× DreamTaq PCR Master Mix (Thermo Scientific) in the presence of a 500 nM mixture of forward and reverse primers (Table 1).

### 2.4. Fluorescence Monitoring in Plants

Fluorescence was monitored in whole plants using a VersaDoc 4000 MP Imaging System (Bio-Rad, Hercules, CA, USA) set with the following parameters: Light mode: LED epi, color: Blue, filter name: 530 BP, gain setting: 1×, and exposure time: 3–120 s.

For microscopy analysis, 2 leaf disks were mounted in water between a slide and cover glass with the upper epidermis forward with a 10× objective. Fluorescence microscopy analysis was performed using a BX53 microscope (Olympus, Shinjuku, Tokyo, Japan) with a GFP-specific filter. Laser-scanning confocal microscopy was performed at the Laboratory of Electron and Confocal Microscopy (Faculty of Biology, Adam Mickiewicz University, Poznań, Poland).

Additionally, fluorescence was measured in a crude extract prepared from plants verified for fluorescence by a DTX 880 Multimode Detector (Beckman Coulter, Brea, CA, USA). For this experiment, 3 disks (5 mm in diameter) from each leaf were sampled and homogenized in 100 µL of sterile water, followed by centrifugation to remove the plant debris. The resulting supernatant was taken for analyses. Fluorescence was measured in a black 96-well plate with a clear bottom using 485/535 nm (excitation/emission) filters.

### 2.5. Immunodetection (IP) of the Recombined GFP Protein

Recombinant GFP was pulled down from ToTV_pJL_-Kra_GFP_-infected plants using IP using GFP-Trap Magnetic Agarose (Chromotek, Planegg-Martinsried, Germany). Briefly, plant material (250–500 mg) was pulverized in liquid nitrogen, followed by homogenization in RIPA buffer (Thermo Scientific). The homogenate was mixed by vortexing for 1 min, followed by centrifugation (14000 rpm for 10 min at 4 °C) to remove plant debris. The resulting supernatant was used for IP.

The GFP-Trap Magnetic Agarose (Chromotek) was gently resuspended (25 µL of the bead slurry per sample) and equilibrated in ice-cold RIPA buffer (Thermo Scientific). The beads were separated with a magnet until the supernatant became clear. The equilibration was performed twice. Next, the lysate was added to the equilibrated beads and mixed end over end for 1 h at 4 °C. The beads were separated with a magnet as mentioned above and washed 3 times with 500 µL of ice-cold RIPA buffer. Finally, the beads were boiled in 50 µL of 2× SDS sample buffer for 5 min at 95 °C to dissociate immunocomplexes.

The recombinant GFP was detected by western blotting, as follows: 30 µL of the protein lysate was fractioned by means of sodium dodecyl sulfate-polyacrylamide gel electrophoresis in a 12% polyacrylamide gel followed by protein transfer onto a PVDF membrane. The filter was blocked for 1 h at room temperature with 5% nonfat milk in phosphate-buffered saline with 0.1% Tween (PBS-T) buffer followed by incubation (1 h at room temperature) with a primary antibody (anti-GFP, Agrisera, Vännäs, Sweden) at a dilution of 1:2000 in blocking buffer. The membrane was washed with PBS-T followed by incubation (1 h at room temperature) with a secondary antibody conjugated with horseradish peroxidase (goat anti-rabbit IgG, Agrisera) at a dilution of 1:10000. After intensive washing, the membrane was developed for 5 min with AgriseraECL SuperBright (Agrisera) solution. Images of the blot were obtained using a CCD imager (VersaDoc 4000 MP Imaging System, Bio-Rad, Hercules, CA, USA). 

## 3. Results

### 3.1. The New Generation of Infectious Clones of ToTV Retains Their Biological Activity

**Figure 1:**
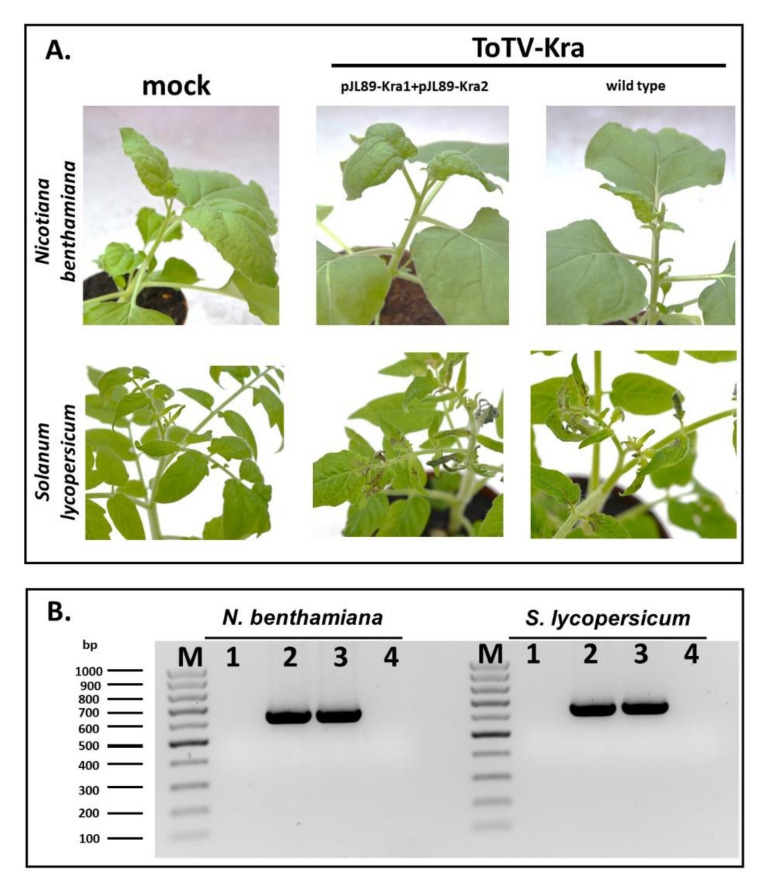
(**A**) Infection symptoms observed in *Nicotiana benthamiana* and *Solanum lycopersicum* (cultivar Betalux) infected with ToTV_pJL_-Kra or wild-type tomato torrado virus (isolate Kra); (**B**) Reverse transcription-polymerase chain reaction (RT-PCR)-based detection of ToTV_pJL_-Kra or wild-type ToTV-Kra in host plants. M-DNA mass ruler, 1—mock-infected plants, 2—ToTV_pJL_-Kra-infected, 3—wild-type ToTV-Kra-infected, 4—no template control; bp- base pairs.

According to our previous observations, the first generation of infectious clones of ToTV [16] performed well in infectivity assays and was successfully used for analyzing ToTV gene functions in the context of virus pathogenicity [14]. However, the clones were found to maintain low stability during their passages in *E. coli* systems (data not shown). Therefore, we found it essential to improve their genetic stability. By performing subcloning procedures aiming at substituting cDNA copies of RNA1 and RNA2 of ToTV-Kra from its original infectious clones (p35Kra1 and p35Kra2), the second generation of plasmids was obtained: pJL89-Kra1 and pJL89-Kra2. The plasmids were used to transform *A. tumefaciens* GV3101 for subsequent agroinfiltration. By performing infectivity assays, it was demonstrated that the mobilized virus, named hereafter ToTV_pJL_-Kra, was infectious to *N. benthamiana* and *S. lycopersicum*. This was confirmed by disease symptoms manifested on agroinfiltrated plants: yellowing and ToTV-specific spoon-like malformations of systemically infected leaves in *N. benthamiana* and leaf mottling followed by severe necrosis developing near veins of systemically infected leaves in *S. lycopersicum* (Figure 1A, middle panel). The same disease symptoms were observed on plants mechanically inoculated with the infectious sap derived from ToTV-Kra-infected *N. benthamiana* (Figure 1A, left panel). The presence of viral RNAs was confirmed in diseased plants utilizing RT-PCR analysis with primers complementary to ToTV RNA2, resulting in the amplification product of the expected size of 624 bp (Figure 1B).

**Figure 2:**
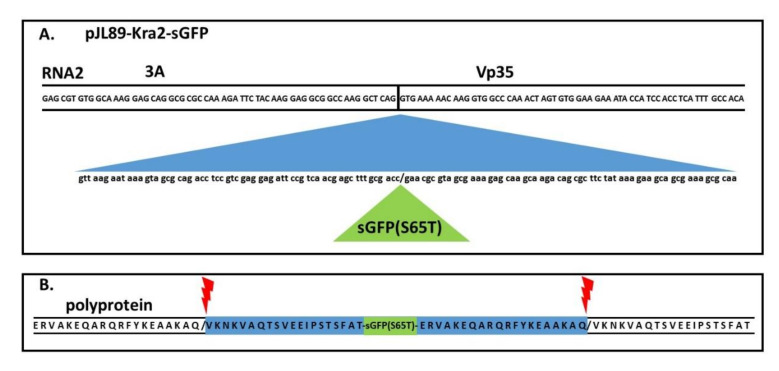
Schematic representation of the modified region of the pJL89-Kra2-GFP infectious clone. (**A**) The putative coding region of the protease recognition site was duplicated (blue) and inserted together with the sGFP (S65T) (green) open reading frame between the 3A/Vp35 junction site; (**B**) The modified virus polyprotein translated from RNA2 with an inserted sGFP open reading frame. The blue region indicates the putative protease recognition site flanking sGFP. The red markers indicate the virus protease cleavage sites at Q/V.

Since it was confirmed that the novel generation of infectious clones of ToTV could infect plants, the pJL89-Kra2 clone was used for further engineering. By performing Gibson assembly, the pJL89-Kra2-sGFP clone was prepared, in which the sGFP open reading frame (ORF, flanked at the N- and C-ends with an additional sequence encoding putative protease recognition sites) was introduced between the 3A and Vp35 coding sequences (Figure 2). The sGFP ORF was inserted seamlessly between 3A and Vp35, as confirmed by Sanger sequencing of the engineered locus within pJL89-Kra2-sGFP. The clone was used to transform *A. tumefaciens* GV3101 bacteria for subsequent agroinfiltration.

### 3.2. GFP-Tagged ToTV Infects N. Benthamiana, Spreads Efficiently in the Host and Can Be Mechanically Passaged

**Figure 3:**
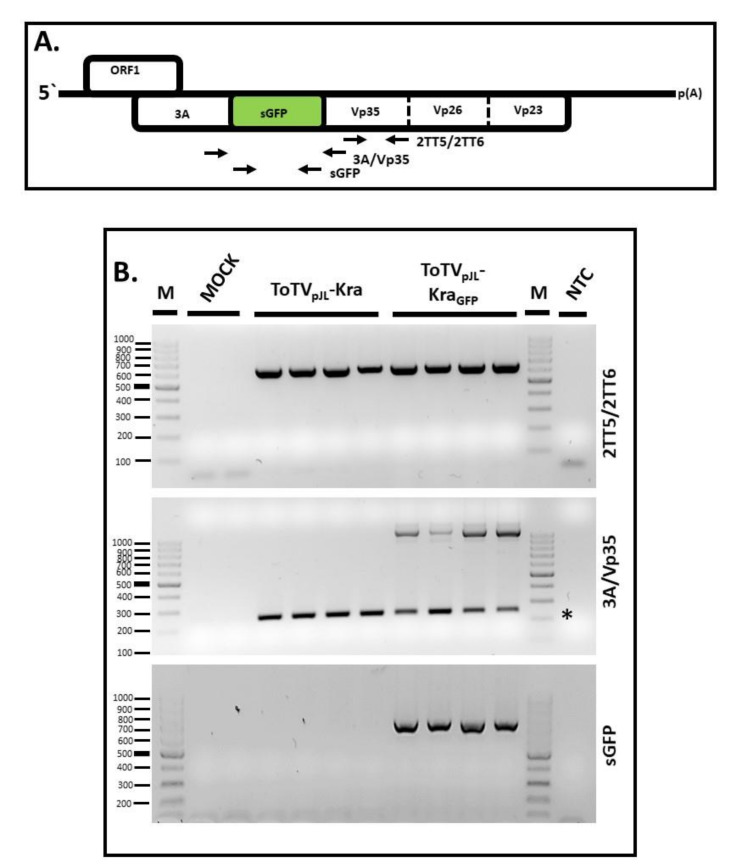
Reverse transcription-polymerase chain reaction (RT-PCR)-based detection of ToTV_pJL_-Kra and ToTV_pJL_-Kra_GFP_ in infected *Nicotiana benthamiana*. Mock-infected plants were also included. (**A**) Schematic representation of the annealing sites of three primer pairs (2TT5/2TT6, 3A/Vp35, and sGFP) used in the RT-PCR detection assays; (**B**) Results of the RT-PCR analysis of the tested plants. The asterisk indicates the amplification products of the locus without sGFP.

To test whether pJL89-ToTV-sGFP can infect host plants, 4 to 6-week-old *N. benthamiana* seedlings were agroinfiltrated with a mixture of *A. tumefaciens* harboring the pJL89-Kra1 and pJL89-Kra2-sGFP clones (forming together in a host into ToTV_pJL_-Kra_GFP_). Six days after infiltration, the plant material was collected and checked for the presence of the engineered virus in their systemic leaves. For this, total RNA was extracted from systemic leaves of tested plants, converted to cDNA, and taken for RT-PCR analysis targeting three loci in RNA2: The Vp35/Vp26 ORF (primers 2TT5/2TT6), the engineered junction site 3A/Vp35 (primers seq3A/Vp35_F/seq3A/Vp35_R) and specifically the sGFP ORF (primers GFP_F/GFP_R) (Figure 3A). RT-PCR with the primers 2TT5/2TT6 resulted in amplification products of 624 bp using RNA extracted from both ToTV_pJL_-Kra and ToTV_pJL_-Kra_GFP_-infected plants (Figure 3A).

In the case of the RT-PCR with primers flanking the 3A/Vp35 engineered region in RNA2, a 249 bp product was expected to be amplified in plants infected by ToTV_pJL_-Kra. Indeed, the amplicon was detected in those plants (Figure 3B). Importantly, the insertion of the sGFP ORF between 3A/Vp35 elongated the tested region by an additional 838 bp. After RT-PCR, the 1087 bp amplification product was detected only in plants infected with ToTV_pJL_-Kra_GFP_. In the same plants, however, an additional RT-PCR product of 249 bp was amplified from the pool of RNA2 lacking the sGFP sequence. Finally, the third RT-PCR performed with the GFP_F/GFP_R primers gave a 717 bp amplification product only in plants infected by ToTV_pJL_-Kra_GFP_ (Figure 3B). All the conducted RT-PCR analyses confirmed that ToTV_pJL_-Kra_GFP_ systemically infected *N. benthamiana*.

Moreover, to test whether ToTV_pJL_-Kra_GFP_ was transmissible from plant to plant, *N. benthamiana* (infected with the modified virus, Supplementary Figure S1) was homogenized, and the obtained sap was used for mechanical inoculation of *N. benthamiana* and *S. lycopersicum* (Betalux cultivar) seedlings. Seven days after inoculation, ToTV_pJL_-Kra_GFP_ was detected in inoculated *N. benthamiana*, as well as in tomato plants (Supplementary Figure S1). These findings were verified by RT-PCR with the aforementioned primer pairs. This result showed that ToTV_pJL_-Kra_GFP_ was infectious and stable through passages in *N. benthamiana* and *S. lycopersicum*.

### 3.3. GFP-Derived Fluorescence is Detected in Plants Infected with ToTV-GFP

**Figure 4:**
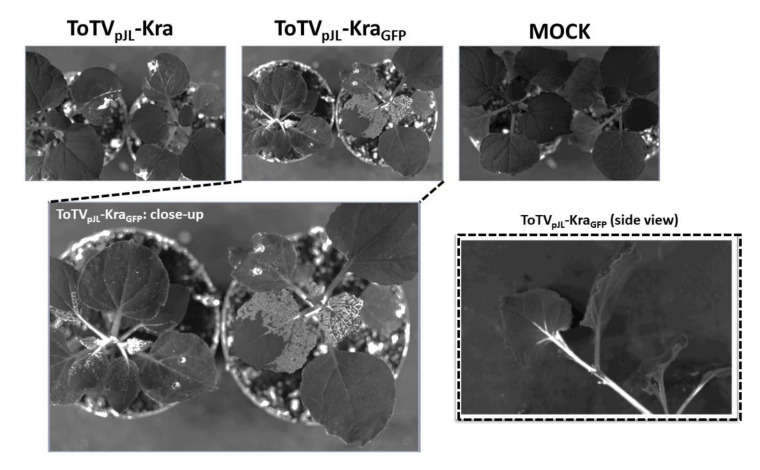
Visualization of sGFP-derived fluorescence in *Nicotiana benthamiana* using a blue LED light source and 530 BP filter. Plants infected with ToTV_pJL_-Kra or ToTV_pJL_-Kra_GFP_ were exposed to a blue LED light source, and fluorescence was detected using a 530 BP filter. Light-gray areas within the systemic leaves of *N. benthamiana* were detected only in plants infected with ToTV_pJL_-Kra_GFP_. The migration of ToTV_pJL_-Kra_GFP_ in the plant (across the main stem, petiole, and primary veins in the leaf) was detected from the side view.

Initially, to verify the expression of sGFP from ToTV_pJL_-Kra_GFP_ in *N. benthamiana*, plants were illuminated under UV using a hand-held lamp. However, under UV light, GFP fluorescence was not detected in ToTV_pJL_-Kra_GFP_-infected plants. Therefore, GFP fluorescence had to be monitored by substantially more sensitive detectors coupled with a CCD camera. Under a blue LED light source and using a 530 BP filter, GFP fluorescence was visualized; within the systemic leaves in plants infected with ToTV_pJL_-Kra_GFP_, fluorescence was monitored and manifested as a strong bright light signal. The signal was detected in veins (vascular tissue) and interveinal areas (mesophyll) of the leaves (Figure 4).

**Figure 5:**
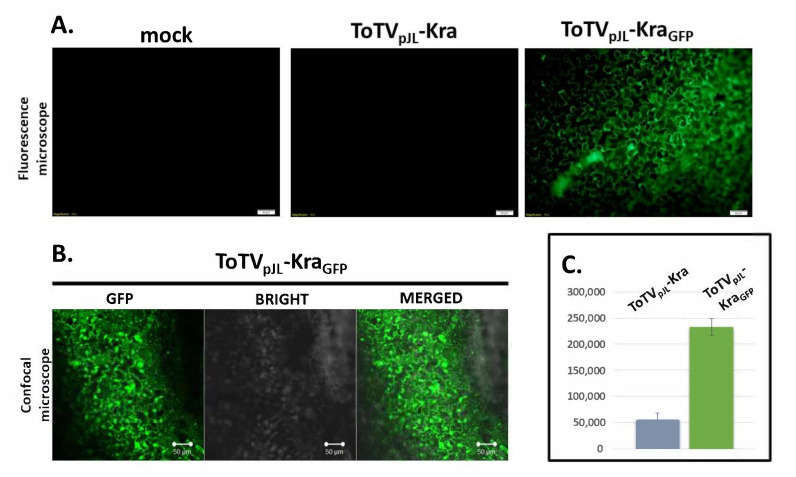
Verification of sGFP-derived fluorescence in *Nicotiana benthamiana* infected with ToTV_pJL_-Kra_GFP_. The analysis was performed using fluorescence (**A**) and confocal microscopy (**B**). Scale bar: Fluorescence microscopy and confocal images = 50 µM; (**C**) Analysis of absolute fluorescence in cleared plant extract of *N. benthamiana* infected by ToTV_pJL_-Kra or ToTV_pJL_-Kra_GFP_.

Importantly, using the same detection system, no fluorescence signal was observed in mock- or ToTV_pJL_-Kra-infected plants. To confirm that the bright light signal was derived specifically from the fluorescence of sGFP, the illuminated leaves were analyzed using a fluorescence microscope. In this analysis, the fluorescence signal observed in the cytoplasm and nucleus was detected only in plant material infected by ToTV_pJL_-Kra_GFP_ (Figure 5A).

Additionally, the plants infected by ToTV_pJL_-Kra_GFP_ were subjected to fluorescence detection by confocal microscopy, and again, the fluorescence signal was confirmed in the plant cells (it was also observed from the nucleus) (Figure 5B). Lastly, the fluorescence level was assessed in cleared leaf extract of *N. benthamiana* infected by ToTV_pJL_-Kra or ToTV_pJL_-Kra_GFP_. Again, this result verified substantially elevated fluorescence levels in plants infected with ToTV carrying the sGFP ORF (Figure 5C). In summary, all the performed analyses confirmed that the described ToTV_pJL_-Kra_GFP_ was capable of infecting the plants, which was accompanied by sGFP-derived fluorescence.

### 3.4. GFP is Produced in N. Benthamiana Infected by ToTV-GFP

**Figure 6:**
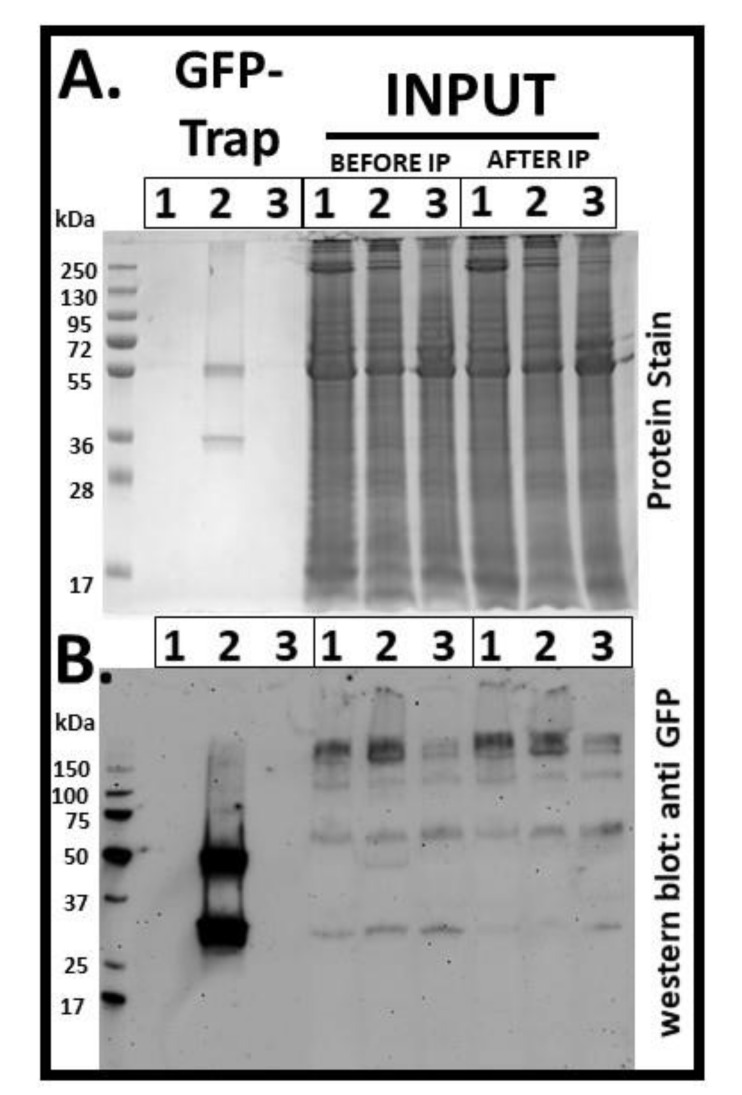
Detection of sGFP accumulation in *Nicotiana benthamiana* infected with ToTV_pJL_-Kra_GFP_ or ToTV_pJL_-Kra. Plant material infected with ToTV_pJL_-Kra (1), ToTV_pJL_-Kra_GFP_ (2), or mock-treated plants (3) were collected and subjected to anti-GFP immunoprecipitation (IP) using GFP-Trap Magnetic Agarose. Protein samples eluted from agarose as well as protein input collected before and after GFP-IP were subjected to sodium dodecyl sulfate-polyacrylamide gel electrophoresis followed by protein staining (**A**) and immunoblot (**B**) analyses. kDa—kilodalton.

To finally confirm that sGFP is produced in ToTV_pJL_-Kra_GFP_-infected plants, immunodetection of the heterologous protein was performed using sGFP-specific antibodies. Preliminarily, western blots were performed using a crude plant extract prepared from *N. benthamiana* infected by ToTV_pJL_-Kra_GFP_. However, no sGFP-specific signal was detected in the assay (data not shown). It was assumed that sGFP accumulated at low levels in plants infected by ToTV_pJL_-Kra_GFP_. Therefore, subsequent western blot assays were performed with sGFP IP followed by immunodetection. Indeed, supported by this approach, the mature sGFP (ca. 30 kDa), as well as polyprotein maturation side products (3A-sGFP and sGFP-Vp35, ca. 49 kDa each), were detected only in plants infected by ToTV_pJL_-Kra_GFP_. sGFP was not detected in the ToTV_pJL_-Kra- or mock-infected plants (Figure 6).

## 4. Discussion

Infectious clones (or infectious transcripts) of plant viruses remain a basic research tool in plant pathology, concerning mostly studies on virus-derived pathogenicity determinants [23]. On the other hand, plant virus vectors were described as platforms for heterologous protein production in plants [24] (revised in [25]) or for silencing gene expression in hosts (most commonly tobacco rattle virus and potato X virus). The goal of this study was to develop stable ToTV-based constructs suitable for expressing a reporter protein, GFP, in plants. During the propagation of the previously described infectious clones p35Kra1 and p35Kra2 [19] in *E. coli*, we found that the constructs could not maintain their stability over time after passaging in the bacteria. This instability manifested with the production of shorter than expected versions of the cloned plasmids after the third round of passaging through *E. coli*. By changing the plasmid backbone (from the original pGreen-based to the pJL89), we observed higher stability of the newly developed infectious clones of the ToTV during their propagation in bacteria. The new generation of infectious clones was able to stably replicate after transferring from low-volume cultures (up to 5 mL) into large-scale ones (up to 300 mL). Indeed, it was described that using low-copy-number plasmids might be beneficial for maintaining the genetic stability of infectious clones [26,27]. Moreover, using the pJL89 plasmid backbone is additionally advantageous because its replication in *A. tumefaciens* does not have to be supported by an additional helper plasmid [28].

In research described by Ferriol et al. [11], a ToMarV isolate M (ToMarV-M, another member of the *Torradovirus* genus) expressing GFP was described. ToMarV-M-GFP was used to verify the self-replicating abilities of RNA1 of the virus and local cell-to-cell movement of the virus. These findings were confirmed by monitoring ToMarV-M-GFP-derived fluorescence, particularly in infiltrated leaves of *N. benthamiana*. In comparison, in our research, we additionally tested the ability for systemic long-distance movement of the recombined virus ToTV_pJL_-Kra_GFP_ within the plant and its ability to infect tobacco and tomato plants via mechanical inoculation. First, we have shown that ToTV_pJL_-Kra_GFP_ can replicate locally and systemically infect *N. benthamiana*. This finding was verified by RT-PCR with sequence-specific primers for ToTV or sGFP. Most importantly, GFP-derived fluorescence, as well as accumulation of the recombinant protein was detected in ToTV_pJL_-Kra_GFP_-infected plants. However, the recombined sGFP could be specifically isolated after subjecting the same extract to a GFP-specific pull-down assay with GFP-Trap Magnetic Agarose. This result might be explained by the high affinity (dissociation constant KD of 1 pM) and binding capacity (8 µg/10 µl of used suspension) of the used affinity resin. ToTV_pJL_-Kra_GFP_ can be used for tracking virus movement in the host, for instance, in studies concerning host- and virus-derived factors determining the pathogen’s host-specific movement [14].

Taken together, all the performed analyses confirmed that heterologous sGFP can be produced in plants using the infectious ToTV_pJL_-Kra_GFP_ clone. More importantly, this clone can be used for monitoring virus cell-to-cell migration as well as long-distance movement in infected plants, which was described here for the first time for the type member of *Torradovirus* genus, in the context of investigating ToTV pathogenicity in the mentioned hosts.

## Figures and Tables

**Table 1:** Primers used in this study.

Primer Name	Sequence 5′→3′	Purpose
pJL89ToT1_R	TATATTCTCAAAATAACTCTTTTAACCTCTCCAAATGAAATGAACTTCC	Amplification of plasmid backbone for cloning of cDNA copy of the ToTV-Kra RNA1
pJL89ToT1i2_F	TTTAAAAAAAAAAAAAAAAAAAAAAAAAGGGTCGGCATGGCATCTC	
pJL89ToT2_R	TATTGTATAAAATTATTCTTTTAAACCTCTCCAAATGAAATGAACTTCC	Amplification of plasmid backbone for cloning of cDNA copy of the ToTV-Kra RNA2
pJL89ToT1i2_F	TTTAAAAAAAAAAAAAAAAAAAAAAAAAGGGTCGGCATGGCATCTC	
asTo1A_pJL_FW	TTTCATTTGGAGAGGTTAAAAGAGTTATTTTGAGAATATAAC	Amplification of cDNA copy of ToTV RNA1
asTo2C_pJL_RV	ATGCCATGCCGACCCTTTTTTTTTTTTTTTTTTTTTTTTTAAAAT	
asTo2A_pJL_FW	TTTCATTTGGAGAGGTTTAAAAGAATAATTTTATACAATATTTATGT	Amplification of cDNA copy of ToTV RNA2
asTo2C_pJL_RV	ATGCCATGCCGACCCTTTTTTTTTTTTTTTTTTTTTTTTTAAAAT	
pJLRNA2_CASF4	AGCAAGACAGCGCTTCTATAAAGAAGCAGCGAAAGCGCAAGTGAAAAACAAGGTGGCCCAAAC	Preparation ToTV-Kra copy of RNA2 variant with duplicated protease recognition site
pJLRNA_CASR4	GGAATCTCCTCGACGGAGGTCTGCGCTACTTTATTCTTAACCTGAGCCTTGGCCGCCTC	
EGFP_CASF3	ACCTCCGTCGAGGAGATTCCGTCAACGAGCTTTGCGACCATGGTGAGCAAGGGCGAG	Amplification of sGFP coding sequence
EGFP_CASR3	TATAGAAGCGCTGTCTTGCTTGCTCTTTCGCTACGCGTTCCTTGTACAGCTCGTCCAT	
2TT5	GATGAGAAAGGAAAGAAGCAG	Reverse transcription-polymerase chain reaction (RT-PCR) for detection of ToTV variants in plants
2TT6	CATATCACCCAAATGCTTCTC	
3A/Vp35seqF	CCCTTTGATTGTTATGATGGCTT	
3A/Vp35seqR	TGGGCCTTACAGCTTCATTG	
GFP_F	ATGGTGAGCAAGGGCGAG	
GFP_R	CTTGTACAGCTCGTCC	

## Supplementary Materials

The following are available online at https://www.mdpi.com/1999-4915/12/10/1195/s1, Figure S1: Green fluorescent protein-tagged ToTV (ToTV_pJL_-Kra_GFP_) is infectious and stable through passages in *Nicotiana benthamiana* and *Solanum lycopersicum*.
