# Supplementary material for: Development of a New Tomato Torrado Virus-Based Vector Tagged with GFP for Monitoring Virus Movement in Plants
Source: Viruses. 2020 Oct 20;12(10):1195. doi: 10.3390/v12101195 (PMC7588970; doi:10.3390/v12101195)
Supplement: Supplementary file 1 [file viruses-12-01195-s001.pdf]

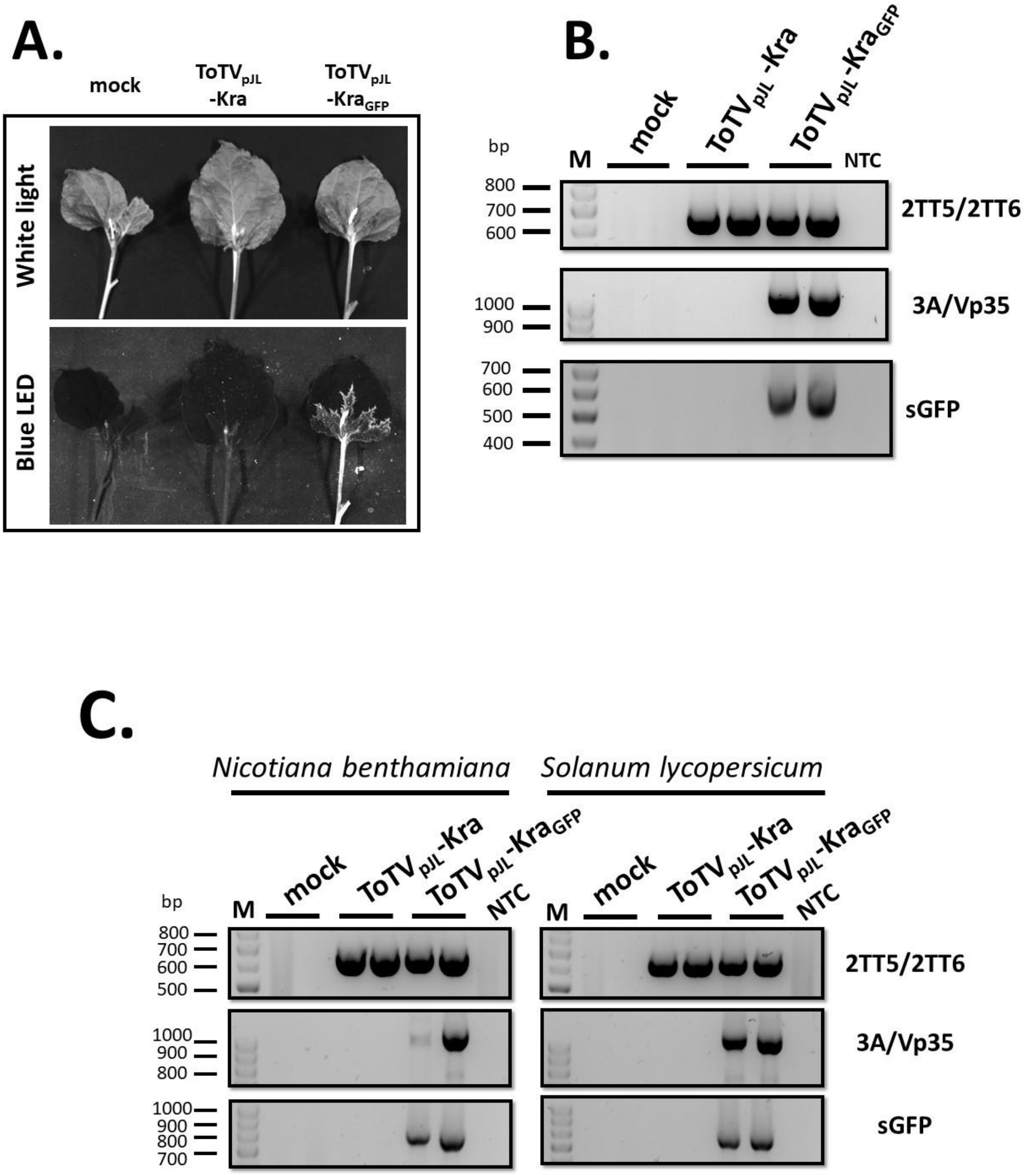

Supplementary Figure S1. Green fluorescent protein-tagged ToTV (ToTV$_{pJL}$-Kra$_{GFP}$) is infectious and stable through passages in *Nicotiana benthamiana* and *Solanum lycopersicum*. (**A**) *N. benthamiana* plants were infected by ToTV$_{pJL}$-Kra or ToTV$_{pJL}$-Kra$_{GFP}$ by agroinfiltration, and six days after infiltration, GFP fluorescence was verified only in ToTV$_{pJL}$-Kra$_{GFP}$-infected plants. Under blue LED light in the leaves and main stems of plants infected by ToTV$_{pJL}$-Kra$_{GFP}$, sGFP-derived fluorescence was detected; (**B**) The

presence of either $ToTV_{pJL}$-Kra or $ToTV_{pJL}$-Kra_{GFP} in *N. benthamiana* was assessed by reverse transcription-polymerase chain reaction (RT-PCR) with three primer pairs (2TT5/2TT6, 3A/Vp35 and sGFP), as described in the Materials and Methods. The positively verified plants were used as virus sources for mechanical sap inoculation of *N. benthamiana* and *S. lycopersicum* seedlings; (**C**) RT-PCR-based analysis of passaged viruses in inoculated *N. benthamiana* and tomato plants. For RT-PCR, the abovementioned primer pairs were used.
